# Treating amyloid transthyretin cardiomyopathy: lessons learned from clinical trials

**DOI:** 10.3389/fcvm.2023.1154594

**Published:** 2023-05-23

**Authors:** Daniela Tomasoni, Giovanni Battista Bonfioli, Alberto Aimo, Marianna Adamo, Marco Canepa, Riccardo M. Inciardi, Carlo Mario Lombardi, Matilde Nardi, Matteo Pagnesi, Mauro Riccardi, Giuseppe Vergaro, Enrico Vizzardi, Michele Emdin, Marco Metra

**Affiliations:** ^1^ Cardiology, ASST Spedali Civili di Brescia, Department of Medical and Surgical Specialties, Radiological Sciences, Public Health, University of Brescia, Brescia, Italy; ^2^ Health Science Interdisciplinary Center, Scuola Superiore Sant’Anna, Pisa, Italy; ^3^ Cardiology and Cardiovascular Medicine Division, Fondazione Toscana Gabriele Monasterio, Pisa, Italy; ^4^ Cardiology Unit, IRCCS OSpedale Policlinico San Martino, Genova, Italy; ^5^ Department of Internal Medicine, University of Genova, Genoa, Italy

**Keywords:** transthyretin cardiac amyloidosis (ATTR-CA), treatment, tafamidis, siRNA, antisense oligonucleotide, gene editing, heart failure

## Abstract

An increasing awareness of the disease, new diagnostic tools and novel therapeutic opportunities have dramatically changed the management of patients with amyloid transthyretin cardiomyopathy (ATTR-CM). Supportive therapies have shown limited benefits, mostly related to diuretics for the relief from signs and symptoms of congestion in patients presenting heart failure (HF). On the other hand, huge advances in specific (disease-modifying) treatments occurred in the last years. Therapies targeting the amyloidogenic cascade include several pharmacological agents that inhibit hepatic synthesis of TTR, stabilize the tetramer, or disrupt fibrils. Tafamidis, a TTR stabilizer that demonstrated to prolong survival and improve quality of life in the ATTR-ACT trial, is currently the only approved drug for patients with ATTR-CM. The small interfering RNA (siRNA) patisiran and the antisense oligonucleotide (ASO) inotersen have been approved for the treatment of patients with hereditary ATTR polyneuropathy regardless of the presence of cardiac involvement, with patisiran also showing preliminary benefits on the cardiac phenotype. Ongoing phase III clinical trials are investigating another siRNA, vutrisiran, and a novel ASO formulation, eplontersen, in patients with ATTR-CM. CRISPR–Cas9 represents a promising strategy of genome editing to obtain a highly effective blockade of *TTR* gene expression.

## 1. Introduction

Amyloidosis is a rare disease caused by the deposition of misfolded fibrillar proteins causing morphological and functional changes in the infiltrated tissues (1). More than 40 different precursor proteins can undergo the substantial molecular transformation to form amyloid fibrils, but most cases of cardiomyopathy (CM) are due to the accumulation of transthyretin (ATTR) or immunoglobulin light chains (AL-CM) (2–4). AL-CM is traditionally considered the most common of these conditions. However, ATTR-CM is increasingly being diagnosed and is emerging as an under-recognized cause of heart failure (HF) in older adults (5, 6). It accounts for 12%–13% of HF with preserved ejection fraction (HFpEF) cases, 5%–7% of patients with presumed hypertrophic cardiomyopathy and 8% of those with severe aortic stenosis (7–10).

Almost all serum TTR is synthesized and secreted in the liver; the choroid plexus and retinal pigment epithelium are other sites of production. Cardiac involvement in ATTR-CM manifests typically as left ventricular thickening and/or HFpEF with increased left ventricular wall thickness and diastolic dysfunction (11, 12). Based on the sequence of the *TTR* gene, ATTR-CM is classified as wild-type (ATTRwt-CM) (without mutation) or mutated/variant (ATTRv-CM) (with a mutation) (5) ATTRwt typically has a late symptom onset (>60 years of age), while symptom onset in patients with ATTRv may occur at younger ages. More than 150 mutations and deletions in the *TTR* gene have been identified (13). The mutations most commonly associated with cardiac involvement are Val122Ile, Thr60Ala, Leu111Met and Ile68Leu (14). Previous studies have shown a prevalence of ATTR-CM in male sex, particularly in ATTRwt. Historically, ATTR-CM phenotype was thought to be less severe in women compared to men, based on non-indexed echocardiographic parameters. A more recent analysis by Patel et al. showed that overall structural and functional phenotype was similar between sexes when indexed to body size, with the only significant differences pointing towards a mildly worse phenotype in females (15). The use of non-indexed parameters could have led not only to underestimate CA severity in female sex, but also to a later diagnosis (age at diagnosis was 3 years higher in females compared to males) and this can partially explain the under-representation of female sex in clinical trials (15, 16).

Recent advances have improved the treatment of ATTR-CM and several clinical trials are ongoing. The aim of our review is to summarize recent findings and future perspectives in the treatment of ATTR-CM.

## 2. Transplantation in ATTR amyloidosis

Until recent times, orthotopic liver transplantation or combined heart-liver transplantation were considered the only available disease-modifying treatments for vATTR amyloidosis (17). Importantly, tissue TTR deposition can progress even after liver transplantation, since TTR amyloid fibres promote subsequent deposition of circulating TTRwt (18). Liver transplantation is not an option for patients with ATTRwt-CM. A few studies conducted on patient with ATTRwt-CM showed excellent outcomes for heart transplantation in highly selected patients, with 92%–100% survival at 3 years and 90% at 5 years (19–21). Risk of allograft amyloid recurrence in short and medium terms appear to be negligible (19).

Due to limited organ availability, exclusion of older patients and of those with advanced systemic disease, the risks related to surgery and life-long immunosuppression, organ transplantation is not suitable for the vast majority of patients with this condition. With the improvement of target therapies in the amyloidogenic cascade, transplantation is not a therapeutic option anymore in many centres.

## 3. ATTR disease-modifying therapies

**Figure 1:**
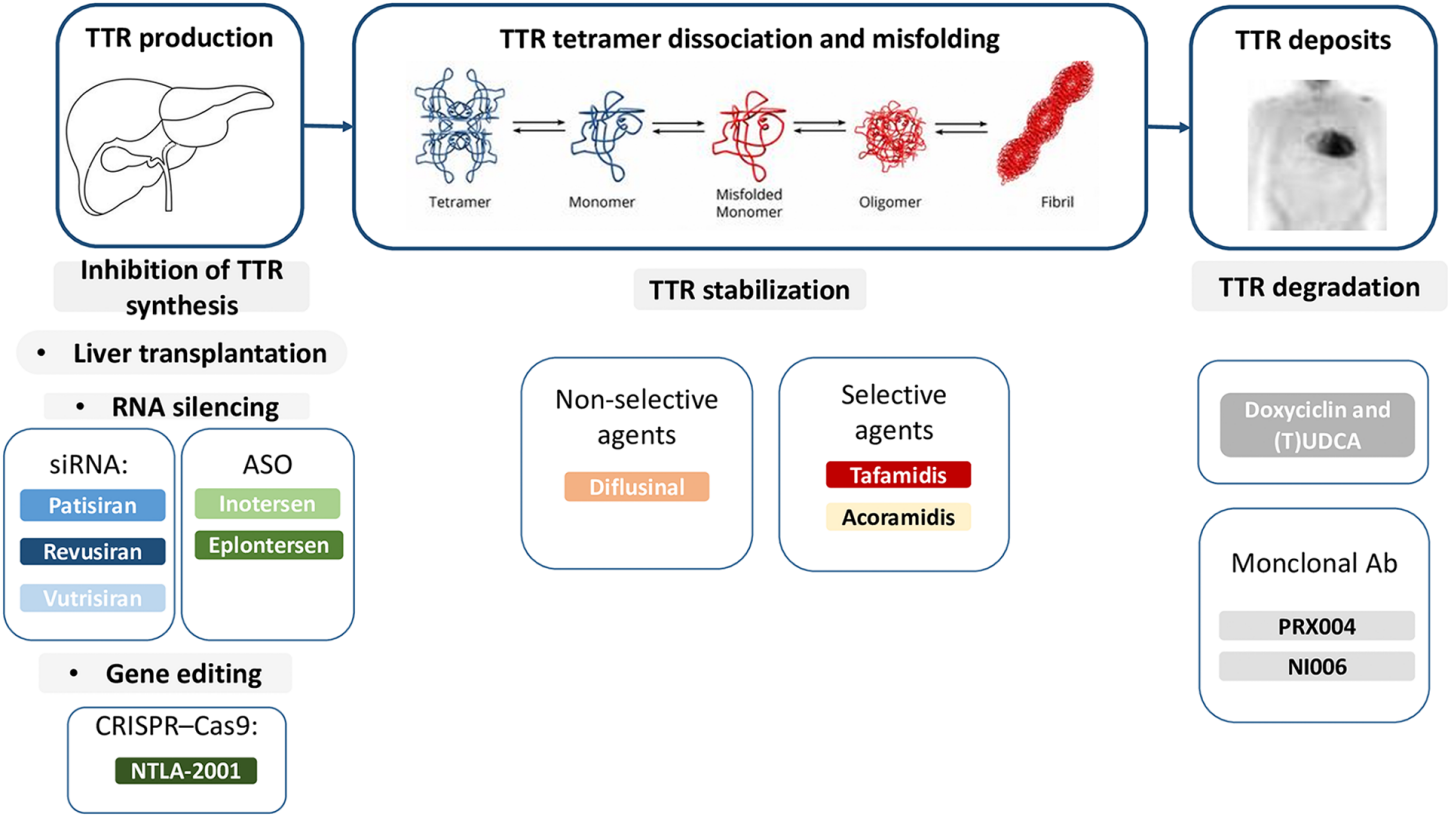
Therapies targeting the amyloidogenic cascade including several pharmacological agents that inhibit hepatic synthesis of TTR, stabilize the tetramer, or disrupt fibrils. Ab, antibodies; ASO, antisense oligonucleotide; siRNA, small interfering RNA; TTR, transthyretin; (T)UDCA, (tauro)ursodeoxycholic acid.

**Figure 2:**
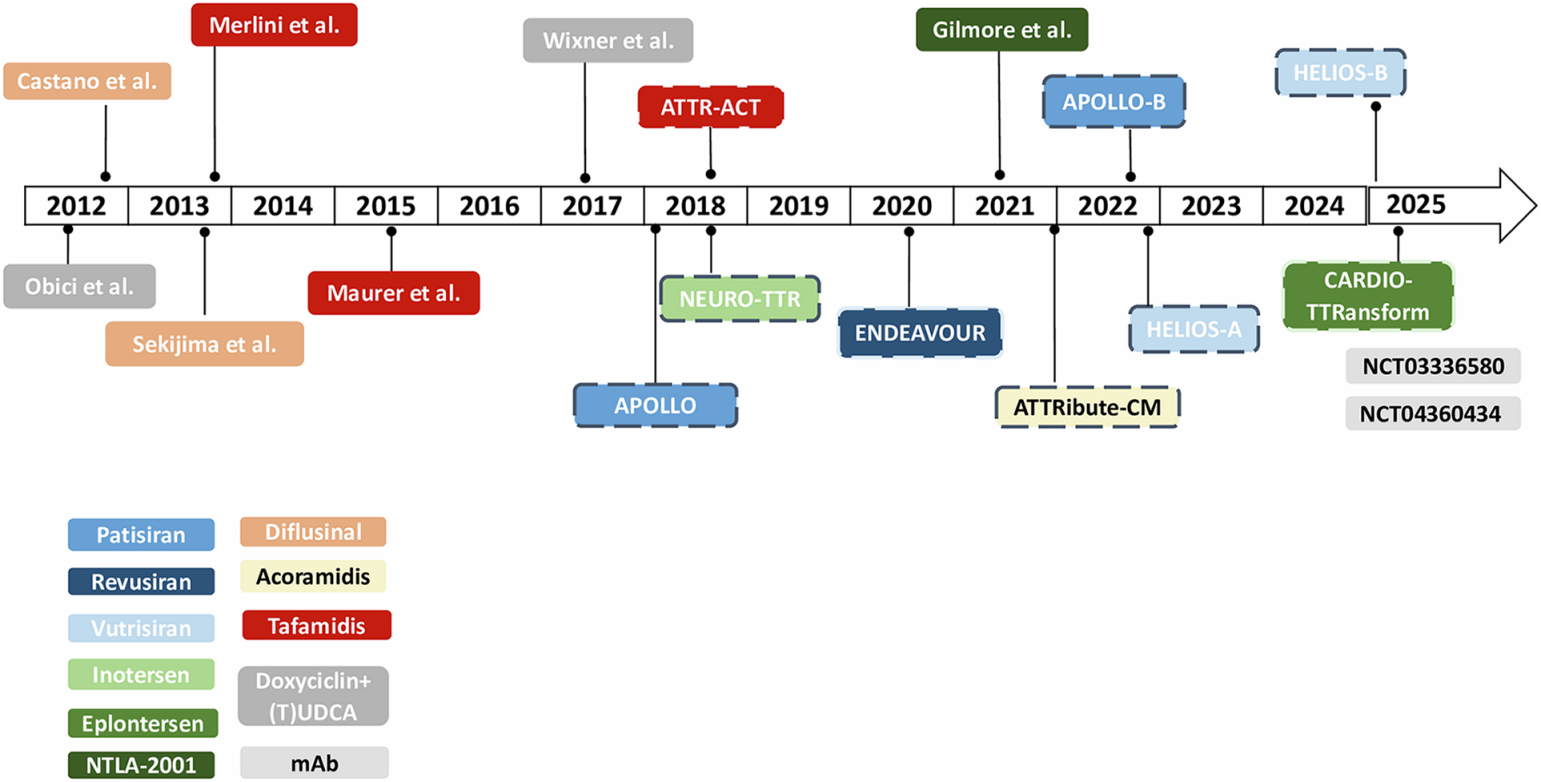
Timeline of clinical trials in ATTR-amyloidosis. *Dotted lines refer to phase III clinical trials. mAb, monoclonal antibodies; (T)UDCA, (tauro)ursodeoxycholic acid.

In the last decades research focused on disease-modifying drugs, that act through different mechanisms: (i) inhibition of amyloidogenic TTR synthesis, (ii) stabilization of the native TTR tetramer structure and (iii) removal of misfolded proteins (Figures 1, 2) (13, 22, 23).

### 3.1. ATTR silencers

No ATTR-silencer is currently approved for the treatment of patients with ATTR-CM, whereas two gene silencers are currently approved for the treatment of patients with ATTRv polyneuropathy, either with or without cardiac involvement: patisiran and inotersen.

#### 3.1.1. Small interfering RNAs

Patisiran is a small RNA-interfering (siRNA) molecule that inhibits hepatic synthesis of TTR by binding RNA silencing complexes (Figure 1). Phase I and II studies showed that patisiran is safe and effective in reducing serum TTR levels (24–26). The APOLLO trial, a randomized, placebo-controlled, phase 3 trial involved 225 patients with ATTRv-polyneuropathy, of whom 126 had concomitant cardiac involvement (27). Patients were randomly assigned in a 2:1 ratio to intravenous patisiran (0.3 mg per kilogram of body weight) or placebo every 3 weeks. Patisiran slowed polyneuropathy progression, assessed using the modified Neuropathy Impairment Score + 7 (mNIS + 7) as primary endpoint, and other measurements including the Norfolk Quality of Life-Diabetic Neuropathy (Norfolk QOL-DN) questionnaire (Table 1) (27). The effects of patisiran on cardiac structure and function were assessed in a pre-specified subpopulation of patients with evidence of cardiac amyloid involvement at baseline (*n* = 126). The pre-specified cardiac subpopulation comprised patients with a baseline left ventricular wall thickness ≥13 mm and no history of hypertension or aortic valve disease. Patisiran reduced mean left ventricular wall thickness (least-squares mean difference ± SEM: −0.9 ± 0.4 mm, *P* = 0.017), improved global longitudinal strain (−1.4 ± 0.6%, *P* = 0.015) and cardiac output (0.38 ± 0.19 L/min, *P* = 0.044), led to increased end-diastolic volume (8.3 ± 3.9 ml, *P* = 0.036) at month 18 and reduced N-terminal pro-B-type natriuretic peptide (NT-proBNP) levels at 9 and 18 months. Moreover, patients in the treatment group had a 46% reduction in the rate of hospitalizations due to cardiovascular (CV) causes and all-cause death compared with those receiving placebo (28). Fontana et al. compared 16 patients with ATTRv amyloidosis treated with patisiran (of whom 12 also received diflunisal) with 16 matched untreated patients with ATTRv amyloidosis. A total of 82% of treated patients showed >80% knockdown in circulating TTR. Patisiran therapy was associated with a reduction in extracellular volume (ECV) {adjusted mean difference between groups: −6.2% [95% confidence interval (CI): −9.5% to −3.0%]; *P* = 0.001} although changes in ECV were highly heterogeneous, with only 6 patients (38%) experiencing an absolute ECV reduction greater than the 3% arbitrary threshold and 3 (19%) showing an increase (38). Patisiran was also associated with a decrease in NT-proBNP concentrations and an increase in 6-minute walking test (6MWT) distances after 12 months of therapy (39). Recently, the positive results of the APOLLO B trial have been announced. The APOLLO-B trial randomized patients with ATTRv- CM or ATTRwt- CM and a history of HF (but current clinically stable), NT- proBNP levels ranging from 300 ng/L to 8,500 ng/L and a 6MWD of ≥150 m to patisiran or placebo. Patisiran met the primary endpoint with a statistically significant improvement in 6MWT compared to placebo at 12 months (−8 m in Patisiran arm vs. −25 m in placebo group, with an overall improvement of 14.7 m with Patisiran compared to placebo). It is questionable whether this improvement might be considered significant in clinical practice. Patisiran also met the first secondary endpoint of improvement in quality of life (QoL) as assessed with Kansas City Cardiomyopathy Questionnaire (KCCQ) (40).

**Table 1:** Therapies for amyloid transthyretin cardiomyopathy (ATTR-CM): evidence from clinical trials.

Drug	Study name (year)	Study design	Population	Outcomes
**TTR silencers**				
SiRNA				
Patisiran	APOLLO (2018) (28, 29)	Phase III, multicentre, randomized, double- blind, placebo- controlled trial; 2:1 randomization to IV patisiran (0.3 mg/kg) or placebo once every 3 weeks for 18 months	225 patients with ATTRv-PN (patisiran *n *= 148; placebo *n *= 77) 126 (56%) patients with concomitant cardiac involvement	Patisiran significantly improved neuropathy scores, QoL, walking parameters, nutritional status and activities of daily living compared to placebo. Patisiran reduced mean LV wall thickness, improved GLS and CO, led to increased LVEDV at month 18 and reduced NtproBNP levels at 9 and 18 months compared to placebo. Reduction of 46% in the rate of CV hospitalization and all-cause death.
	APOLLO-B (2022) (27)	Phase III, randomized, double-blind, placebo-controlled multicenter (patisiran vs placebo)	Patients with ATTRv- or ATTRwt-CM and history of HF; NT- proBNP ranging from 300 ng/L to 8,500 ng/L; 6MWD ≥150 m	Patisiran significantly improved 6MWT and QoL, assessed by KCCQ, at 12 months.
Revusiran	ENDEAVOUR (2020) (30)	Phase III, multicentre, randomized, double-Blind, placebo-controlled; 2:1 randomization to SC revusiran (500 mg) or placebo daily for 5 days, then weekly for 18 months	206 patients with ATTRv- CM (revusiran *n *= 140; placebo *n *= 66)	NA (trial stopped early due to increased mortality compared with placebo
Vutrisiran	HELIOS-A (2022) (31)	Phase III, multicentre, randomized, open- label; 3:1 randomization to SC vutrisiran (25 mg) once every 3 months or IV patisiran (0.3 mg/kg) once every 3 weeks, for 18 months	164 patients with ATTRv- PN (vutrisiran *n *= 122; patisiran *n *= 42)	Vutrisiran signficantly improved neuropathy scores and QoL at 9 months
	HELIOS-B	Phase III, multicentre, randomized, double- blind, placebo- controlled; SC vutrisiran (25 mg) or placebo once every 3 months	Patients with ATTRv- CM or ATTRwt- CM	Estimated study completion date 2025
ASO				
Inotersen	NEURO-TTR (2018) (29)	Phase III trial, randomized, double-blind, placebo controlled; 2:1 randomization to weekly subcutaneous injections of inotersen (300 mg) or placebo.	172 patients with stage 1–2 ATTRv- PN (inotersen *n *= 112; placebo *n *= 60) 108 (63%) patients had ATTRv-CM	Inotersen improved the course of neurologic disease and QoL. No differences in global longitudinal strain and other echocardiographic variables at 15 months Several adverse events, including severe events such as glomerulonephritis and thrombocytopenia, causing durg discontinuation.
Eplontersen	CARDIO-TTRansform	Phase III, multicentre, randomized, double- blind, placebo- controlled trial; randomization to SC injections of either eplontersen or placebo once every 4 weeks	Patients with ATTR-CM (estimated 1400 participants)	Estimated study completion date 2025
Gene editing—CRISPR-Cas9				
NTLA-2001	Gillmore et al. (2021) (32)	Phase I, multicentre, randomized, open- label, placebo- controlled; single IV dose of NTLA-2001: 0.1 mg/kg (*n *= 3) or 0.3 mg/kg (*n *= 3)	6 patients with ATTRv-PN	Interim results from the first two single- dose groups of the trial: administration of NTLA-2001 showed a 52% (47%–56%) in patient receiving 0.1 mg/kg and 87% (80%–96%) in patient receiving 0.3 mg/kg reduction in serum TTR protein concentration from baseline
TTR stabilizers				
Diflunisal	Castano et al. (2012) (33)	Single-arm, open-label study; oral diflunisal 250 mg b.i.d.	13 patients with ATTRv-CM or ATTRwt-CM	Administration was safe, even if not associated with significant change in cardiac structure, function or biomarkers
	Sekijima et al. (2013) (34)	Single-centre study; Diflunisal was administered orally at 500 mg/day	40 patients with ATTRv amyloidosis	Diflunisal was well tolerated and stabilized TTR tetramer
Tafamidis	ATTR-ACT (2018) (35)	Phase III, multicentre, double blind, placebo-controlled trial; 2:1:2 randomization to oral tafamidis 80 mg daily, tafamidis 20 mg daily, or placebo for 30 months	441 patients with ATTRv-CM or ATTRwt-CM (pooled tafamidis: n = 264; placebo: *n *= 177)	Tafamidis was associated with lower all-cause mortality than placebo (HR, 0.70; 95% CI, 0.51–0.96) and a lower rate of cardiovascular related hospitalizations, with a relative risk ratio of 0.68 (0.48 per year vs. 0.70 per year; 95% CI, 0.56–0.81). Differences in overall survival emerged after approximately 18 months. Tafamidis showed also benefits on 6MWT and KCCQ-OS, already relevant at 6 months.
Acoramidis (AG10)	ATTRibute-CM	Phase III, randomized, double-blind, placebo-controlled study; randomization acoramidis (AG10) 800 mg administered orally twice a day vs placebo	Patients with ATTRv-CM or ATTRwt-CM and history of HF	Acoramidis did not change 6MWD at month 12 compared to placebo. An improvement in KCCQ-OS and a decline in Nt-proBNP concentrations as well as in serum TTR concentrations were observed.
TTR disrupter				
Doxycicline and TUDCA	Obici et al. (2012) (36)	Phase II, open-label study with doxycycline (100 mg twice/day) and TUDCA (250 mg three times/day) administered continuously for 12 months	20 patients with TTR-amyloidosis (both FAP or CM)	Doxycicline and TUDCA stabilized the disease for at least 1 year. NIS-LL: stable; LV wall thickness: stable or reduced; NYHA class: no change; NT-proBNP: stable or increased; Improvement of SF-36 physical and mental scores
Doxycicline and UDCA	Wixner et al. (2017) (37)	Phase II study; Part 1: 12-month doxycycline 200 mg/day for 4 weeks and UDCA 750 mg/day with intermittent discontinuation for 2 weeks; Part 2 (6 months): no treatment.	28 patients with ATTR-CM	High discontinuation rate due to treatment failure, side effects and voluntary dropouts. NT-proBNP: no change at month 6; increase at month 12 No increase in LV septum thickness at month 12
Monoclonal antibodies (mAb)				
PRX004	NCT03336580	Phase I study	Subjects with ATTR amyloidosis	Ongoing
NI006	NCT04360434	Phase I study	Patients with hereditary or wild-type ATTR-CM	Ongoing

Revusiran is a siRNA that was investigated in the phase III ENDEAVOUR trial, enrolling patients with ATTRv-CM. The trial was stopped early due to the high rates of death (13% of patients receiving revusiran and 3% of those receiving placebo) recorded during a median follow-up of 7 months. The majority of patients enrolled in the trial died because of HF. A *post hoc* safety investigation of patients treated with revusiran found that a greater proportion of those who died had ≥75 years and more advanced HF, although a role of revusiran could not be excluded (41).

Vutrisiran is a second-generation siRNA targeting TTR mRNA64 similar to patisiran, with an enhanced stabilization chemistry that allows its subcutaneous administration every 3 months. An initial phase I study proved its safety and efficacy (42). The subsequent HELIOS-A trial included 164 patients with ATTRv-PN who received a 25 mg dose of vutrisiran every 3 months (*n* = 122) or active comparator patisiran (*n* = 42). At 9 months, vutrisiran met the primary endpoint of improvement in mNIS +7 score and of all secondary endpoints (31). Vutrisiran is being studied for the treatment of both ATTRv and ATTRwt-CM in the phase III, randomized, double- blind, placebo- controlled, multicentre HELIOS-B trial. The primary endpoint is a composite of all- cause mortality and recurrent CV events (CV hospitalizations and urgent HF visits) at 30–36 months (NCT04153149) (Table 1). Full results are expected in early 2024.

#### 3.1.2. Antisense oligonucleotides

Antisense oligonucleotide (ASO) can silence target mRNA sequences by a variety of mechanisms, mostly involving degradation mediated by the endogenous ribonuclease RNase H1. Inotersen is a 2′-O-methoxyethyl–modified ASO, that binds the 3′ untranslated region of human TTR mRNA (both wildtype and variant) and inhibits the production of liver TTR-protein (Figure 1). It is administered weekly by subcutaneous injection. Inotersen was initially tested in a phase I, randomized, placebo- controlled, double-blind, dose-escalation study with patients receiving the highest dose regimen showing the greatest reduction in serum TTR levels up to 76% (43).

The NEURO-TTR was a randomized, double-blind, placebo controlled, phase 3 trial that randomized 172 patients in a 2:1 ratio to weekly subcutaneous injections of inotersen (300 mg) or placebo. Inotersen improved the course of neurologic disease and QoL (Table 1). However, several adverse events occurred in the treatment group (e.g., injection site reactions, nausea, headache, fatigue, fever and thrombocytopenia) causing drug discontinuation in 14% of the cases. Furthermore, serious adverse events as glomerulonephritis (3%) and thrombocytopenia (3%, with one consequent death) were registered. Thus, frequent platelet count and renal function monitoring is recommended during treatment (29). This important adverse effects might result in treatment cessation, and have significant implications on the use of this drug in both current clinical practice and in the setting of a clinical trial. In the subgroup of patients with cardiac disease (as well as in the whole population) from the NEURO-TTR trial no significant differences were reported regarding echocardiographic parameters at the 15-month follow-up (29). A small single-centre, open-label study including patients affected by ATTRwt-CM and NYHA I-III showed, at a 2- and 3-years follow-up, a reduction in left ventricular (LV) mass measured by magnetic resonance imaging (MRI) and an increase in exercise tolerance as measured by 6MWT. The main adverse effect was inflammation and induration associated with subcutaneous injection; a few patients developed generalised “flu-like” symptoms 24–48 h after the injection, at first or second exposure and without further episodes. At 3 years, the average platelet count was decreased by 15% but none of the patients had bleeding episodes or severe thrombocytopenia (44). A further study is ongoing (NCT03702829).

Eplontersen is a novel antisense nucleotide that is administered subcutaneously every 4 weeks and with no serious adverse effects reported in a phase I study. NEURO-TTRansform is a phase III trial that randomized 140 patient with stage 1 or 2 hereditary transthyretin-mediated amyloid polyneuropathy (ATTRv-PN) in a 6:1 ratio to either eplontersen 45 mg once every 4 weeks or inotersen 300 mg once a week until the pre-specified week 35 interim efficacy analysis. Participants received daily supplemental doses of the recommended daily allowance of vitamin A. Participants included in the inotersen reference arm were crossed over to eplontersen at Week 37 after completing the Week 35 assessments. The final efficacy analysis at week 66 compared eplontersen with the historical placebo arm from NEURO-TTR trial (45). At 66 weeks, patients treated with eplontersen demonstrated a statistically significant and clinically meaningful change from baseline vs. placebo group on the co-primary endpoints of modified Neuropathy Impairment Score +7 (mNIS + 7) and Norfolk Quality of Life Questionnaire-Diabetic Neuropathy (Norfolk QoL-DN). The trial also met its third co-primary endpoint demonstrating a statistically significant reduction in serum TTR concentration in the eplontersen vs. placebo group. TTR reductions were consistent with those reported in the interim analysis at week 35. Eplontersen' efficacy in ATTR-CM is being studied by CARDIO-TTRansform study that will randomize 1,400 patients with ATTR-CM and NYHA class I-III to eplontersen (subcutaneous injection once every 4 weeks) or placebo. The primary endpoint is a composite of CV mortality or recurrent CV clinical events up to week 140. Secondary outcome will evaluate also functional capacity and QoL. (NCT04136171).

### 3.2. ATTR stabilizers

Tetramer stabilizers prevent monomer misfolding and deposition by binding to the T4-binding site on TTR (e.g., tafamidis and diflunisal) or by mimicking the super-stabilizing TTR variant T119M (e.g., acoramidis).

#### 3.2.1. Non-selective agents: diflunisal

Diflunisal is a non-steroidal anti-inflammatory (NSAID) drug that can be classified as a non-selective ATTR stabilizer agent. In 2006, a phase 1 study showed that diflunisal (250 mg per os b.i.d.) complexes to the T4 binding site and stabilizes TTR tetramers, thus preventing amyloid fibril formation *in vitro*. In a single-arm, open-label study (mean follow-up 0.9 ± 0.3 years) involving 13 patients with confirmed ATTRwt- or v-CM, diflunisal was safe, but did not improve cardiac structure, function, or biomarkers (33). In a further study including 40 ATTRv amyloidosis patients, diflunisal stabilized TTR tetramer structure over a mean follow-up of 38.0 ± 31.2 months. As a NSAID, the main adverse effects include gastrointestinal bleeding and renal failure (34).

#### 3.2.2. Selective stabilizers: tafamidis, acoramidis

Tafamidis is a small molecule that stabilizes TTR tetrameric structure by binding to the protein's thyroxine binding sites. In the early 2010s tafamidis (at a dosage of 20 mg) proved effective and safe for the treatment of patients with early-stage Val30Met transthyretin familial amyloidotic polyneuropathy (FAP) with a reduction in the neurological symptoms' progression (46). The results were then confirmed in a phase 2 open-label, single-treatment arm study enrolling patients with non-Val30Met TTR amyloidosis (47).

Tafamidis was firstly approved by the European Medicines Agency (EMA) for the treatment of TTR amyloidosis in adult patients with stage 1 symptomatic polyneuropathy but not by the US Food and Drug Administration (FDA).

The ATTR-ACT trial, published in September 2018, was a multicentre, international, double-blind, placebo-controlled, phase 3 trial that randomized 441 patients with ATTR-CM (both variant and wt) in a 2:1:2 ratio to tafamidis 80 mg, 20 mg or placebo. The primary outcome was a hierarchical composite of all-cause mortality, followed by frequency of cardiovascular-related hospitalizations. Over a 30-month follow-up the rates of the primary endpoint were lower among patients in the treatment arm compared to those who received placebo (*P* < 0.001). Tafamidis was associated with both lower all-cause mortality [hazard ratio, 0.70; 95% confidence interval (CI), 0.51–0.96] and a lower rate of CV-related hospitalizations than placebo, with a relative risk ratio of 0.68 (0.48 per year vs. 0.70 per year; 95% CI, 0.56–0.81) (48). Differences in overall survival emerged after approximately 18 months. Secondary endpoints included performance on 6MWT and KCCQ-Overall Summary (KCCQ-OS) score. The benefits of tafamidis on these secondary endpoints were already evident at 6 months (48). Long-term extension study with all patients subsequently switched to the highest dose supported tafamidis 80 mg (bioequivalent of tafamidis free acid 61 mg) as the optimal dose, considering the lack of adverse events and a significant reduction (30%) in the relative risk of death on a median follow-up of 51 months (30). Among patients enrolled in the ATTR-ACT trial, 335 were affected by ATTRwt-CM and 106 by ATTRv-CM. Patients with ATTRwt had milder disease and lower rate of disease progression over the study. However, tafamidis showed similar benefits on mortality and QoL in both subgroups (49). A *post hoc* analysis of ATTR-ACT reported a reduction in patients treated with tafamidis of both CV hospitalization (−32%) and mean length of stay per CV-related hospitalization events. Taken together, tafamidis reduced by 2.62 days CV-related hospitalizations per patient per year and in a subgroups analysis of patients with NYHA functional class I or II, by 3.96 days (50). Earlier treatment initiation was associated with better outcomes. Indeed, an interim analysis of the long-term extension to the pivotal ATTR-ACT showed that patients initially treated with tafamidis in ATTR-ACT had substantially lower mortality than those first treated with placebo and then transitioned to tafamidis [79 (44.9%) deaths with continuous tafamidis and 111 (62.7%) with placebo to tafamidis, hazard ratio, 0.59 (95% CI, 0.44–0.79); *P* < 0.001] (51). The reduction in mortality was similar in patients with ATTRwt and patients with ATTRv (≈40% in each), whereas there was a greater reduction in patients with NYHA class I or II (44%) than NYHA class III (35%) in the continuous tafamidis group compared with the placebo to tafamidis group (51).

Based on the results of the ATTR-ACT trial and of these subgroup analyses showing greater benefits in patients with NYHA functional class I and II, the 2021 ESC guidelines for the diagnosis and treatment of acute and chronic heart failure recommend tafamidis for the treatment of patients with ATTRv or ATTRwt cardiomyopathy, and NYHA class I or II to reduce symptoms, CV hospitalization and mortality (class of recommendation I, level of evidence B) (3). The 2022 ACC/AHA/HFSA guidelines recommend tafamidis also in patients with NYHA class III symptoms and add that tafamidis should be considered in select patients (52).

Acoramidis, also known as AG10, binds with high affinity and selectivity TTR in human serum. It is designed to mimic the structure of the protective T119M mutation and forms hydrogen bonds with the same serine residues at position 117 that stabilize the T119M variant. It has proven efficacy in stabilizing TTR both for Val122Ile mutation and TTRwt amyloidosis without significant adverse effects (53). Acoramidis 800 mg twice a day vs. placebo is currently being investigated in ATTRibute-CM, a phase 3 randomized controlled trial enrolling patients with symptomatic ATTR-CM (NCT03860935). ATTRibute-CM did not meet its primary endpoint at month 12 with a mean observed 6MWD decline of 9 meters and 7 meters for the acoramidis and placebo arms, respectively. However, an improvement in KCCQ-OS and a decline in Nt-proBNP concentrations as well as in serum TTR concentrations were observed (54). Considering that tafamidis showed a significant reduction in mortality only after 18 months, the investigators included a co-primary endpoint in a longer timeframe, a hierarchical combination of all-cause mortality, frequency of cardiovascular-related hospitalization and change in the total distance walked in 6 min over a 30 months period.

### 3.3. TTR disruption/resorption

As both TTR stabilizers and silencers do not influence advanced ATTR-CMs, drugs defined as ATTR degraders are supposed to be the only ones that may lead to pathology regression, targeting already deposited amyloid fibrils.

Doxycycline proved able to remove TTR amyloid deposits, and tauroursodeoxycholic acid (TUDCA) reduced non fibrillar-TTR and other markers associated with pre-fibrillar TTR (Figure 1). Combined administration of these drugs had synergic effects in removing amyloid deposits in transgenic TTR mice models, in early disease stages (55). A phase II, open-label study demonstrated that doxycycline (100 mg twice/day) and TUDCA (250 mg three times/day) administered continuously for 12 months in patient with TTR-amyloidosis (FAP and/or CM) stabilized the disease for at least 1 year. Only 2 patients out of 20 discontinued the therapy, one due to gastric pain and the other because of persistent nausea and loss of appetite (36). Another phase II study conducted on 28 patients with ATTR-CM with a combination of doxycycline and ursodeoxycholic acid (UDCA), showed less benefits and a high discontinuation rate due to treatment failure, side effects and voluntary dropouts. The study was divided into two parts, the first one with a 12-month period treatment with doxycycline 200 mg/day for 4 weeks with intermittent discontinuation for 2 weeks, associated with UDCA 750 mg/day; the second consisted in a withdrawal period in which disease progression was monitored. The high rate of treatment failure may indicate that doxycycline discontinuation may decrease the efficacy of the drugs and that TUDCA may be more effective than UDCA (37). However, the results remain difficult to be interpreted (22). ATTR degraders showed better results in younger patients with less advanced disease (56). Doxycycline plus TUDCA or UDCA are currently not approved by EMA or FDA due to the small number of patients included in the studies and the controversial results (57). Wider RCTs are needed to assess effectiveness of the treatment.

### 3.4. Gene editing

Clustered regularly interspaced short palindromic repeats and associated Cas9 endonuclease (CRISPR-CAS-9), enabling targeted *in vivo* genome editing, could be an emerging therapy in ATTR amyloidosis. A recent report described the partial results of a phase I, open-label, multicentre trial on an *in vivo* gene-editing therapeutic agent, called NTLA-2001, showing promising results. NTLA-2001 is based on the CRISPR-Cas9 system and comprises a lipid nanoparticle encapsulating messenger RNA for Cas9 protein and a single guide RNA targeting TTR. This preliminary analysis was conducted on 6 patients with ATTRv-PN. NTLA-2001 was administered on a single escalating dose of 0.1 or 0.3 mg per kilogram. To avoid inflammatory reactions patients were pre- treated with glucocorticoids and antihistamines. Twenty-eight days after the injection, mean reduction in serum TTR protein concentration from baseline was 52% with 0.1 mg/Kg and 87% with 0.3 mg/Kg. Therapy was well tolerated and no patients experienced serious adverse events (32). Recruiting in the study is still ongoing with a target of 72 patients (NCT04601051). This study will evaluate safety, tolerability, pharmacokinetics and pharmacodynamics of NTLA-2001 in ATTRv-PN and in both ATTRv-CM and ATTRwt-CM. Estimated primary completion date is March 2025. NTLA-2001 has a clear advantage compared to siRNA-based and ASO-based therapies since the knockdown is expected to be permanent after a single injection not requiring serial infusions.

### 3.5. Monoclonal antibodies

In the last years anti-TTR humanized monoclonal antibodies (mAb) were also investigated (Figure 2) (13). In preclinical studies on ex vivo and *in vivo* models these antibodies could promote clearance of amyloid fibrils by selectively targeting ATTR aggregates and promoting macrophage-mediated phagocytosis. Importantly, these antibodies did not react with native TTR tetramers but detected misfolded proteins, by targeting epitopes more exposed in TTR dissociative monomers, non-native oligomers, and/or aggregates (58, 59).

PRX004 is an investigational mAb designed to prevent fibril formation by specifically targeting and clearing the misfolded forms of the TTR protein found in ATTR-CM. Its safety, tolerability, pharmacokinetics, pharmacodynamics, and maximum tolerated dose are currently being evaluated in a phase 1 study (NCT03336580). NI006 is another investigational human mAb that targets TTR amyloid. A phase 1 trial with NI006 evaluating its safety, pharmacokinetic profile and exploratory outcome measures of efficacy in patients with hereditary or wild-type ATTR-CM is ongoing (NCT04360434) (13).

## 4. ATTR symptomatic treatment

### 4.1. Heart failure

The main goal of symptomatic therapy in patients with amyloid CM is to improve QoL and well-being. The supportive treatment in patients with signs or symptoms of HF is based on maintenance of euvolaemia and consists in loop diuretics (usually furosemide) (3, 60). The optimal balance between diuretic therapy and fluid status may be challenging to achieve due to the reduced stroke volume typical of restrictive cardiomyopathies (61).

The use of neurohormonal modulators, namely beta-blockers, angiotensin converting enzime inhibitors (ACE-Is), angiotensin receptor blockers (ARBs) and angiotensin receptor neprilysin inhibitor (ARNI) is controversial in those patients presenting with HF and reduced ejection fraction (62). Indeed, on one hand patients with CA may have a significant neurohormonal activation similarly to non-amyloidotic HF patients (63). On the other hand, these drugs may be not tolerated due to the development of hypotension (62, 64). Beta-blockers may be poorly tolerated also because of conduction disturbances or decreasing cardiac output with consequent exercise intolerance when cardiac output becomes critically dependent on heart rate (35, 65). In a recent study including 309 consecutive patients with ATTR-CM, there was no association of neurohormonal blockade use with survival (64). In the 2021 ESC position paper on the diagnosis and treatment of cardiac amyloidosis, it is recommended to avoid beta-blocker and ACE-i/ARBs as they exacerbate hypotension, particularly when amyloid autonomic dysfunction is present (2). Similar indications can be found in other major documents from scientific societies on cardiac amyloidosis (60). Nevertheless, about one third of patients in the ATTR-ACT trial were on beta-blockers or ACEi/ARB (48).

Conversely, use of MRA is not generally contraindicated and it is recommended (Class I) in Canadian Cardiovascular Society/Canadian Heart Failure Society (CCS/CHFS) cardiac amyloidosis' guidelines even in the absence of specific studies (66). MRAs are already approved for the treatment of HFrEF (Class I) and HFmrEF (Class IIb) (3) and recent studies focused on the use of spironolactone in HFpEF. Treatment of Preserved Cardiac Function Heart Failure With an Aldosterone Antagonist (TOPCAT) is a phase III, multicenter, international, randomized, double-blind, placebo controlled trial conducted on 3,445 patients that showed that adding spironolactone to existing therapy in patient with HF and LVEF ≥45% did not significantly reduce the incidence of the primary outcome of death from CV causes, aborted cardiac arrest or hospitalization for HF. However, HF hospitalizations were significantly reduced in the spironolactone group. An analysis from TOPCAT trial showed that the subset of patients with structural and functional echocardiographic features typical of cardiac amyloidosis, despite having a worse prognosis, experienced similar benefits from spironolactone therapy to other patients (67).

The diagnosis of CA was an exclusion criterion in major clinical trials on sodium-glucose-cotransporter-2 (SGLT2) inhibitors. A series of 15 consecutive patients with ATTR-CA and diabetes treated with SGLT2 inhibitors by the diabetologists has been reported without significant adverse effects (68). Due to their cardiorenal benefits, the robust safety and tolerability, with clinical trial data reporting minimal effects on blood pressure, glycaemia-related adverse events, and no excess in acute kidney injury (69, 70), it appears reasonable that SGLT2 inhibitors may be well tolerated and might be helpful, at least due to their add-on diuretic effect and by reducing diuretic resistance. Currently, no RCTs specifically investigated safety and benefits of HF therapies, namely neurohormonal antagonists, SGLT2 inhibitors, in patients with cardiac amyloidosis.

Similarly, the oral soluble guanylate cyclase stimulator vericiguat may be considered in patients with HFrEF, NYHA class II-IV who have had worsening HF despite other evidence based medical therapies in patients with HFrEF (71). However, CA was an exclusion criteria of the VICTORIA trial and vericiguat has never been tested in patients with ATTR-CM.

### 4.2. Atrial fibrillation and anticoagulation

Atrial fibrillation (AF) seems to be more common in cardiac amyloidosis than in the general population. Sanchis et al. reported an overall AF prevalence of 44% among patients with both AL and ATTR-CM (72). The prevalence is even higher in patients with ATTRwt-CM and has been estimated around 70% (73–75). However, previous studies have shown no relationship between AF and prognosis in ATTR-CM (72, 73). This might be explained by the frequent absence of atrial contraction at echocardiographic assessment, defined as “atrial electromechanical dissociation” (AEMD) even in sinus rhythm (SR) (76). These patients presenting AEMD have, indeed, a poorer prognosis than CA patients in SR but with an effective atrial contraction and have similar prognosis to those with AF (76).

As a consequence of atrial dysfunction and enlargement, patients with ATTR-CA have a higher risk of left atrial thrombosis even when in SR. This may also be related to the amyloid deposition into the atrial wall, and the cardiotoxic damage of atrial cardiomyocytes by amyloid precursors (77). Among patients with CA and AF/atrial flutter, anticoagulation is indicated regardless of CHA2DS2-VASc score as in these patients it is not associated with the probability of LAA thrombosis. The role of anticoagulation in patients with CA and sinus rhythm as well as the choice between vitamin K antagonists (VKAs) and direct oral oral anticoagulants (DOACs) are other unmet needs that should require a RCT. A few retrospective studies showed no differences between DOACs and VKAs in embolic events, bleeding risk and overall mortality (78–80). A study by Mentias et al. analysed 551 patients with amyloidosis and new diagnosis of AF and found a decreased risk of mortality, ischemic stroke and major bleeding events when DOAC was prescribed, compared to VKA (81). Transesophageal echocardiogram should be performed in all patients before cardioversion of atrial arrhythmias since the chance of intracardial thrombus remains high, even among patients receiving adequate anticoagulation (82, 83).

Management of AF in cardiac amyloidosis is complicated due to restrictive physiology and the frequent association with autonomic dysfunction. Patients often do not tolerate rate control therapies (beta-blockers, calcium-channel blockers and digoxin) as they may exacerbate hypotension and they reduce the compensatory heart rate, which is the main driver of cardiac output (84). As a result, rhythm control strategy may be preferred. However, Mints et al. found no mortality benefit with antiarrhythmic drugs vs. rate control strategy (73). Evidence regarding transcatheter ablation of atrial arrythmias are limited to date. El-Am et al. investigated outcome of direct-current cardioversion (DCCV) for atrial arrhythmias in patients with CA. Although the success rate of restoring sinus rhythm was high, tachyarrhythmias and bradyarrhythmias complicating DCCV were significantly more frequent in CA patients compared with control patients (82). In all these studies, the rate of AF recurrence was higher than in the general population. More recently, Donnellan et al. has shown that rhythm control strategies, including antiarrhythmic drugs, ablation and DCCV, were more effective when performed early in the course of the disease (85).

### 4.3. Rhythm disturbances and devices

As a consequence of myocardial tissue infiltration, ATTR-CM patients are more prone to develop rhythm disturbances, such as atrio-ventricular blocks, sick sinus syndrome or atrial fibrillation with bradycardia, needing pacemaker implantation (2, 7). Predictors of pacemaker implantation have been recently described in a large cohort of patients (19). The indications for pacemaker implantation are the same as in patients without CA in the absence of specific evidence (60).

As ATTR-CM is a restrictive cardiomyopathy, stroke volume may be markedly reduced and chronic right ventricular apical pacing can result in left ventricular dyssynchrony. Cardiac resynchronisation therapy (CRT) may be considered if a high burden of pacing is expected (5, 61). Nonetheless, the benefits of CRT have been established in patients with non-amyloidotic HF and further investigations are warranted in CA. An implantable cardioverter defibrillator (ICD) should be used in secondary prevention with standard indications (60). ICD implantation in primary prevention is controversial as sudden cardiac death may be caused by electromechanical dissociation as seen in studies conducted mainly on AL-CM patients. Nevertheless, selected patient may benefit from ICD placement (22, 60, 86–88).

### 4.4. Aortic stenosis

Among patients with proven TTR-CA, up to 16% had moderate or severe aortic stenosis (AS). ATTR-CM has a prevalence ranging from 4% to 29% among patients with severe AS, with a higher prevalence of the low-flow low-gradient phenotype (89–92). Notably, amyloid deposition did not worsen prognosis of patients undergoing transcatheter aortic valve replacement (TAVR) (90, 91). Further studies are needed to assess the best treatment options in these patients.

## 5. Final remarks

Increased awareness in ATTR-CM has led to the development of new disease-modifying therapies, to an earlier diagnosis in the course of the disease and to a better management of the patients. (93) Ioannou et al. performed a retrospective analysis using data from the National Amyloidosis Centre, London, between 2002 and 2021 with the aim to characterize changes in the clinical phenotype of patients diagnosed with ATTR-CA over the past 20 years. They showed a progressive increase in the referrals. This was accompanied by a greater number of ATTR-CA diagnoses (*n* = 35 in the first period, 2002–2006; *n* = 968 in the last period, 2017–2021), predominantly of the wild-type form. Importantly, a greater proportion of patients were diagnosed at an early-stage over time. This was associated with a progressive decrease in mortality during the study period (2007–2011 vs. 2012–2016: hazard ratio, 1.57 [95% CI, 1.31–1.89], *P* < 0.001; and 2012–2016 vs. 2017–2021: hazard ratio, 1.89 [95% CI, 1.55–2.30], *P* < 0.001) (94).

Patients in earlier milder disease stages are less likely to experience events (death, heart failure admissions, arrhythmia) in clinical trials. This might explain why the ATTR-ACT trial was successful (as patients enrolled some years ago may have been sicker, with the placebo group experiencing more events), whereas the more recent ATTRibute-CM testing acoramidis failed its primary endpoint at 12 months. Of note, a mortality benefit with tafamidis was only seen at 18 months, as opposed to 12 months, which again might in part explain the failure of AG10 to meet primary end point at 12 months. As a result of the above, there is now a need of wider RCTs. While the ATTR-ACT trial enrolled <500 patients, more recent studies had to extend the population included. The ongoing eplontersen' CARDIO-TTRansform has significantly expanded its recruitment target. The initial recruitment target was below 1,000, but the trial sponsors have recognised the importance of the changing phenotype over time and have rightfully opted to expand recruitment significantly to 1,400 in an attempt to increase the power of the study. The earlier diagnosis of the disease, the discovery of an appropriate therapy and the consequent reduction in overall mortality can explain the negative results of some recent trials.

Nowadays, the only approved treatment for ATTR-CM is tafamidis, a TTR stabilizer, that has shown important benefits on survival and QoL in the ATTR-ACT trial. Tafamidis exceeds conventional cost- effectiveness thresholds. The high cost of tafamidis prevents certain patients from accessing treatment, particularly in privatised healthcare systems. Furthermore, the high cost has led to tafamidis not being publicly funded in countries with publicly funded free healthcare. Such countries include UK and Australia. This high cost barrier to these patients accessing life changing medication is extremely important, and really emphasises the need for stringent, thoughtful and appropriately powered clinical trial design so that ultimately patients can get access to affordable treatment.

Of note, as the first benefits of tafamidis on prognosis have been shown after 18 months of treatment, it should be started soon in the natural course of the disease, therefore an early diagnosis remains crucial. Two TTR silencers are approved only for patients with ATTRv polyneuropathy and possible concomitant cardiomyopathy: patisiran and inotersen. TTR silencers and stabilizers prevent amyloid formation but have no effects on already deposited fibrils. TTR degraders can remove fibrils and bring a regression of the pathology but have more adverse effects and no effective phase III trial was yet conducted. Hypothetically, an association between stabilizers/silencers and degraders may be a good strategy, but there is no clear evidence yet. New anti-TTR humanized antibodies could be an alternative in fibrils removal, mediated by phagocyte activation. CRIPSR-CAS9, an innovating gene-editing therapy used in hereditary pathologies could be a real revolution in the natural course of ATTR-CM with first studies showing promising results.

Symptomatic therapies, except for diuretic therapy in decompensated HF, are largely not assessed. ACEi/ARB/ARNI and beta-blockers are generally not recommended even in patients with reduced LVEF. Conversely, spironolactone showed a potential benefit in patients enrolled in the TOPCAT trial with suspected CA. SGLT2 inhibitors deserve further investigations. Anticoagulation in patients in both sinus rhythm and atrial fibrillation remain a major unmet need.
